# Physical Necessities

**Published:** 1893-03

**Authors:** 


### HALL'S JOURNAL OF HEALTH

TRUTH DEMANDS NO SACRIFICE; ERROR CAN MAKE NONE.

Vol. 40. MARCH, 1893. No. 3.

### PHYSICAL NECESSITIES.

Since the decease of so many of our prominent men, in such nearness to each other in point of time, a great deal has been written and published concerning their physical and mental habits, and no little speculation indulged in relative to the causes of their untimely taking off. Mr. Blaine, as we all know, was a hard worker even in private station, and as Secretary of State, his handling of public affairs in the crises of international disturbances, demanded his undivided attention, weighting him heavily with responsibilities inseparable from his office, wherein a single misstep or a blunder might have entailed a national calamity. Whilst his mental capacity was prodigious and kept constantly at a severe tension, his bodily requirements were neglected until it was too late to restore the necessary equilibrium, without which, good health is a thing impossible. The resignation of his office was not followed by that recuperative rest which nature demanded as a condition of future well being.

The leading habit of General Butler was unceasing activity. Ever since the close of the civil war, in which he bore a soldier's part, the requirements of his various professional engagements, involving the gravest interests, have kept him constantly on the go, from one judicial tribunal to another in rapid succession. For him there was no rest. No sooner had he dealt a final blow in the arena of legal combat in one place, than he turned up in another with only a change of armor. As his physical endurance diminished, he resorted to external aids without abandoning the field or laying aside the work-a-day harness, as if the end were far away, and died with the harness on.

We know less about the habits of General Doubleday, whose name has passed into history as a brave and able officer devotedly attached to the Union cause in the ever memorable struggle for national unity.

Bishop Brooks, of the Massachusetts diocese, passed to the other life in the very prime of manhood, beloved by all who had the good fortune to know him. What has been said of him and his work in various secular and denominational organs since his untimely taking off forms a united testimonial to the greatness and goodness of the man in his chosen field of labor, as by a single voice. Men of this stamp constitute the moral wealth of the nation and their lives are too precious to be sacrificed to the demon of overwork.

Jay Gould was a victim to intense mental and physical exertion and overstrain. His unceasing activity, as the organizer and director of great enterprises, undermined a constitution naturally robust and capable of more than ordinary endurance. He toyed with railroads as children toy with games, organizing detached lines into systems that spanned a continent as by a single thread, but it would seem with the sole object of enriching himself, and this he accomplished in a time and to an extent unparalleled in the records of human achievement, and paid the penalty of overwork in an enfeebled constitution and premature death. His unexpected end was a shock to the financial world but beyond that, it made no sound and caused no ripple upon the level waters of common-place affairs. He will be missed in Wall street, but scarcely anywhere else, for he bequeathed of his millions, nothing to charity.

None of these men, so eminent in their respective spheres, died of old age, but rather from the habitual abuse of faculties and organs governed by laws as strict and immutable as is a tree, a flower, or a machine formed by the ingenuity of man. The tree must be fertilized or perish, and the machine continually lubricated or break down, and it is equally so with brain and muscle and nerves.

It is seldom that one, in this haram-scaram age, dies a purely natural death, for a natural death follows upon natural life, and that is next to impossible in view of the artificial ways which fashion and elegance require in a state of what is deemed the highest civilization. The savage, who roams the forest and sleeps in the open air, subsisting upon fruit and nuts and such game as his ingenuity is able to entrap, comes nearer to it than he who dwells in a palace, sleeping in a close room and living upon rich and indigestible food.

We not only require a plain, simple diet served at regular periods, but we should partake of it deliberately and with good cheer. The way of gulping food common to most people, is a sin against one of the simplest rules of health, and it is equally wrong to rush from the table to the workshop, the library or the counting house. There should be a period of rest, if only for a half hour after meals, followed by moderate exercise. Our hours of sleep should be regular and sufficient, and that too, in a well ventilated room.

We all need and must have rest—rest from thinking, rest from labor, rest from everything that wastes the tissues and sends the worn out cells to the surface. Give nature time and opportunity to recuperate her forces and restore her relaxed energies, and she will do it in her own way. You cannot force her to it in your way, or do something else in the same time. She is jealous of her rights and will not yield them however urgent the demand upon her. The whole subject may be summarized thus: Pure air, pure water, healthy food, proper exercise, sufficient rest, undisturbed hours of sleep and suitable clothing. All these without overwork of brain or body, and the rest will care for itself.

